# Whole genome diversity of inherited chromosomally integrated HHV-6 derived from healthy individuals of diverse geographic origin

**DOI:** 10.1038/s41598-018-21645-x

**Published:** 2018-02-22

**Authors:** Marco Telford, Arcadi Navarro, Gabriel Santpere

**Affiliations:** 1 Institute of Evolutionary Biology (UPF-CSIC), Departament de Ciències Experimentals i la Salut, Universitat Pompeu Fabra, PRBB, Barcelona, Catalonia Spain; 2 National Institute for Bioinformatics (INB), PRBB, Barcelona, Catalonia Spain; 3 Institució Catalana de Recerca i Estudis Avançats (ICREA), PRBB, Barcelona, Catalonia Spain; 4 Center for Genomic Regulation (CRG), PRBB, Barcelona, Catalonia Spain; 5 Department of Neuroscience, Yale School of Medicine, New Haven, CT 06510 USA

## Abstract

Human herpesviruses 6-A and -B (HHV-6A, HHV-6B) are ubiquitous in human populations worldwide. These viruses have been associated with several diseases such as multiple sclerosis, Hodgkin’s lymphoma or encephalitis. Despite of the need to understand the genetic diversity and geographic stratification of these viruses, the availability of complete viral sequences from different populations is still limited. Here, we present nine new inherited chromosomally integrated HHV-6 sequences from diverse geographical origin which were generated through target DNA enrichment on lymphoblastoid cell lines derived from healthy individuals. Integration with available HHV-6 sequences allowed the assessment of HHV-6A and -6B phylogeny, patterns of recombination and signatures of natural selection. Analysis of the intra-species variability showed differences between A and B diversity levels and revealed that the HHV-6B reference (Z29) is an uncommon sequence, suggesting the need for an alternative reference sequence. Signs of geographical variation are present and more defined in HHV-6A, while they appear partly masked by recombination in HHV-6B. Finally, we conducted a scan for signatures of selection in protein coding genes that yielded at least 6 genes (4 and 2 respectively for the A and B species) showing significant evidence for accelerated evolution, and 1 gene showing evidence of positive selection in HHV-6A.

## Introduction

Human herpesvirus 6 (HHV-6) is a globally dispersed dsDNA virus that infects more than 90% of the adult human population^1–3^. HHV-6 presents two species (HHV-6A and HHV-6B) with high nucleotide identity^4,5^ but with different epidemiology, biology and immunologic profiles^6^.

HHV-6 achieves latency by integration at the subtelomeric end of the telomeres of very few host cells^7^, making it arduous to detect in the absence of primary infection or reactivation^8,9^. While the preferential replication site for HHV-6 are CD4^+^T lymphocytes *in vitro*^10^ and *in vivo*^11^, they are characterized by a broad tropism for human cells^12–15^. The genomes of HHV-6A and -6B are similarly organized, with most of the genes encoded in a long non-repetitive region, enclosed between two identical long repeats (ca 8 Kb) called DR_L_ and DR_R_. The two DRs are flanked by additional repeats (T1 and T2) composed of perfect telomere-like repeats (TTAGGG)n in variable copy number. The T1 region is longer than T2, and presents as well degenerate telomere-repeats^16,17^. These telomere-like repeat allow for homologous recombination with the subtelomeric end of the telomere region^7^, and has been found in different chromosomes, but usually in a single copy per host^7–9,18–26^. While both T1 and T2 take part in the integration process, only T2 has been shown to be necessary^16^.

The integration in the telomere region could have negative effects on the host cell as it could interfere with its protective role against chromosome shortening or incorrect identification of the chromosome end as a double-strand break^27,28^. Huang *et al*. showed that while telomeres *in vitro* (lymphoblastoid cell lines (LCL)) presenting HHV-6 integration were shorter than average, *in vivo* (sperm DNA) showed no different sign of erosion^29^. Nevertheless, the impact of HHV-6’s integration on telomere functionality and on the host cell is yet to be fully understood and other aspects unrelated to chromosome shortening might be affected^30^. Integrated HHV-6 has been shown to be in instances able to fully reactivate *in vitro*^7,31^ and *in vivo*^32–34^.

The integration occasionally occurs in germinal cells allowing it to enter the germ line. The virus can then be transmitted vertically in subsequent generations and be present in every cell of the offspring^33^ as a congenital condition. This condition is classically referred to as inherited chromosomally integrated HHV-6 (iciHHV-6) or endogenous HHV-6, and it affects 0.2–1% of the world adult population^20,33,35,36^. The most common, non-congenital, form of the virus is referred to as exogenous HHV-6. The congenital form of the virus can consist of an intact genome, which can potentially lead to the expression of the whole viral gene set within every cell of the carrier^7,37^. The implication of the presence of iciHHV-6 and its relation with HHV-6-related diseases are currently being explored^24,26,29,38^.

While only the HHV-6B species is a recognized etiological factor for *exanthema subitum*^39^, both species are linked to viral fever, febrile seizure^40^, graft rejection^41,42^, and are common causes of *status epilecticus*^43^. Both species present a wide range of putative disease associations, with different degree of characterization and support. Among these we find multiple sclerosis (MS)^44–46^, Hodgkin’s lymphoma (HL)^23,26,47,48^, and encephalitis/meningitis (EM)^49–51^.

The study of the pathological association of HHV-6 is limited by its high seroprevalence and detection rates that hamper the determination of causal links between virus and diseases. Also, there are numerous confounding variables that can influence the interpretation of results or mask an association, primarily their variability, their putative geographical stratification and the differences between exogenous and endogenous HHV-6. To estimate these factors, a much more complete set of sequences is needed. Both prevalence of HHV-6 and HHV-6-associated diseases show worldwide geographical variation^52–57^. For instance, MS has higher prevalence in high-latitude and Caucasian populations^58^, while HL and non-HL have higher incidence in Europe and North America compared to Africa, Asia and South America^59^.

Genetic differences among HHV-6 strains could contribute to these marked geographical patterns in epidemiology of the above-mentioned diseases. However, and due to the scarcity of full HHV-6 sequences, little is known about the genetic stratification among HHV-6 viruses in latency within individuals from different populations.

We performed a systematic search for the presence of HHV-6 within individuals from different populations sequenced in the 1000 Genome Project^60^ (1KGP), a large-scale database of whole human genomes from healthy individuals (http://www.internationalgenome.org/). We identified 11 individuals presenting HHV-6 in a possible congenital state from which we could obtain total DNA of derived LCLs. We then performed target enrichment of the entire HHV-6 genome, allowing us to reconstruct 9 almost complete genomic sequences. Through droplet digital PCR we quantified the virus copy number, supporting the congenital state of the viruses, producing a new data set of iciHHV-6. We carried out a comprehensive comparative analysis across HHV-6 genomes to reveal the geographical structure of genetic diversity, the patterns of recombination along the virus’ genome and putative targets of positive selection in protein coding genes.

## Results

### A small proportion of 1KGP individuals show HHV-6 presence

The detection of the whole HHV-6 genome using reads from the 1KGP low-coverage dataset (mean depth = 7,4x^61^) would be a solid sign of the presence of latent integrated HHV-6. Out of 2,535 individuals scanned from the 1KGP, 11 showed higher number of reads mapping to HHV-6, which amounts to 0.44% of the whole data set.

**Figure 1:**
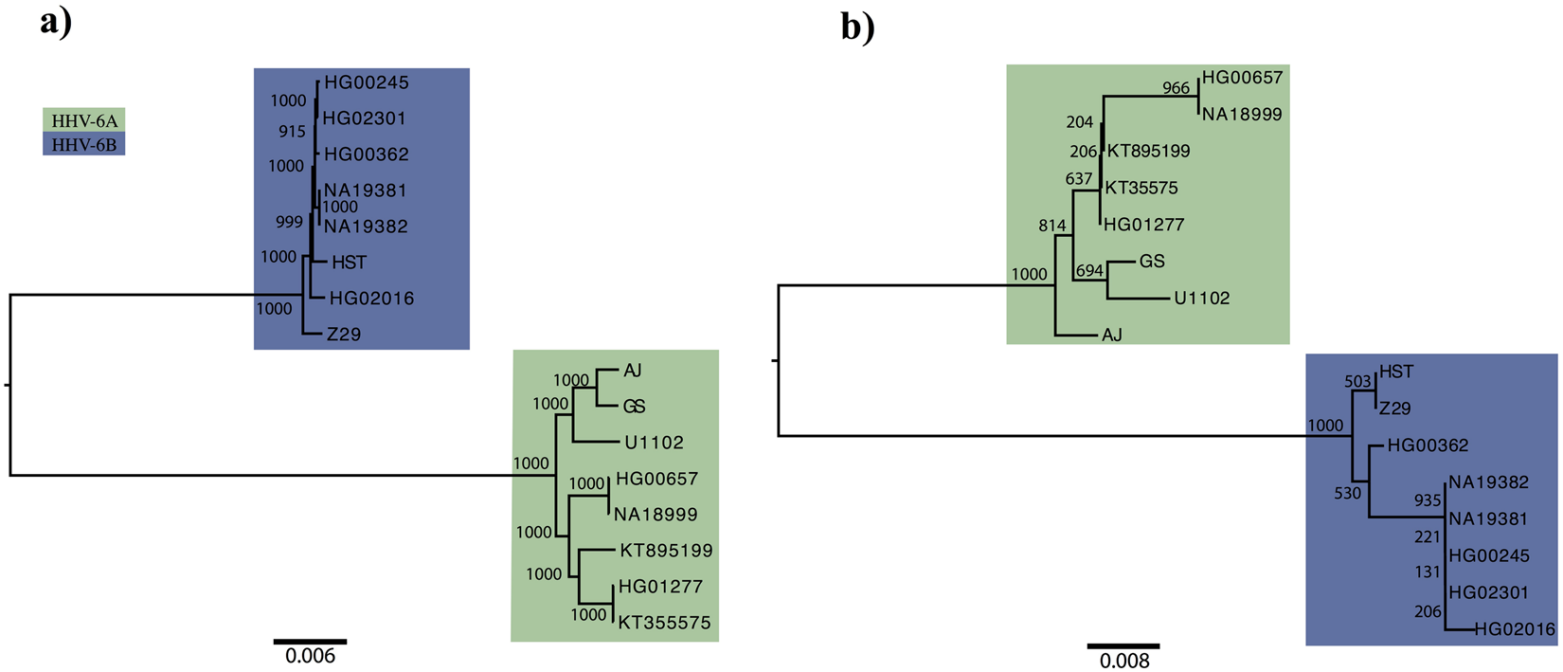
Species assignation through Neighbor-joining tree. The trees were built using the whole HHV-6 data set (**a**), and using only the U83 ORF (**b**). The different virus species are colour-coded, showing the clear separation between HHV-6A and HHV-6B strains. U83-based tree presents lower bootstraps due to the low number of variants present in the limited U83 region.

We searched for signs of the presence of a single or multiple species within one sample by two approaches: a) counting uniquely mapping reads and density of mismatches against both species. b) constructing phylogenetic trees to provide bootstrap support for the separation between species using the whole genome or using only the U83 ORF. U83 is the most divergent ORF between HHV-6A and HHV-6B^4,5,62^. We observed a clear pattern of mismatches discriminating both species and a clear separation between HHV-6A and –B with maximum bootstrap support (Fig. 1). We concluded that in all HHV-6 positive individuals only a single HHV-6 species (Table 1) was detected.

**Table 1:** Median viral coverage before and after target enrichment. Results for HHV-6 mapping performed on the 1000 Genome Project selected individuals (on the left), and on the sequences generated through target enrichment. On the left the length of the complete virus genome covered after quality filters is shown as percentage of the whole genome. Proportion of mapping reads between the species for which there is a higher number of mapping reads (dominant) and the alternative one (alternative) is also shown. On the right the median values for the whole genome are shown for both species. First-degree family members are marked in italic.

Individual	Sample origin	1000 GP low-coverage data			Target enrichment data	
		Covered Genome (%)	Infecting Variant	Infecting/ Alternative	HHV-6A	HHV-6B
iciHG00245	**UK**	**56**	B	1.68	12	**125**
iciHG00362	**Finland**	**72**	B	2.04	15	**150**
iciHG01058	**Puerto Rico**	**42**	B	2.04	Nd*	Nd*
iciHG01162	**Puerto Rico**	**62**	B	1.98	Nd*	Nd*
iciHG02016	**Viet Nam**	**58**	B	2.06	18	**159**
iciHG02301	**Peru**	**80**	B	1.92	13	**141**
*iciNA19381*	**Kenya**	**62**	B	2.04	11	**89**
*iciNA19382*	**Kenya**	**69**	B	1.97	9	**94**
iciHG00657	**China**	**50**	A	1.86	**229**	33
iciHG01277	**Colombia**	**68**	A	1.81	**140**	16
iciNA18999	**Japan**	**61**	A	1.70	**77**	9

*No data due to unavailability of biological sample for target enrichment.

### Target enrichment produces high coverage HHV-6 genomes

We obtained total DNA from the LCL from the 9 of the 11 HHV-6-positive individuals which were available from Coriell Institute for Medical Research. We performed target enrichment of the viral genome using baits designed to capture the two species of HHV-6. Captured DNA was multiplexed and sequenced on a single Illumina MiSeq System flow-cell. Reads from each sample were mapped against the two HHV-6’s species references. Median coverages obtained ranged from 77x to 229x. Lastly, we checked for species-specific Single Nucleotide Variants (SNVs) within the U65 gene for both HHV-6A and HHV-6B^63^, confirming in all cases our assignment predictions, and the absence of the two species in a single individual.

To avoid ambiguities in variant calling, we masked long repeats and low-complexity regions (>500 bp), which resulted in the masking of the two long terminal Direct Repeats DR_L_ and DR_R_ (left and right), and the internal ones R1, R2 and R3 in both species, plus R0 for the B species (Repeats coordinates shown in Supplementary Table S1). That left a total of 138,144 bp and 140,628 bp for HHV-6A and HHV-6B respectively (~87% of the whole genome for both species) for further analysis.

Sequences were then scanned for large structural variation, but no signs of duplications, inversions or large insertions/deletions were found (Supplementary Figure 2).

We further masked base pairs with low depth of coverage or extreme allele balance for each sample (see “Materials and method section”). In order to do comparative analyses, we calculated the “comparable region”, also known as common “callable” region, defined as all the bases of the virome that were covered after the quality filters by all individuals in the data set. The resulting comparable region showed lowly fragmented genomes (135 fragments for A and 94 for B) that account for 83.0% of the HHV-6B complete genome (95.2% of the repeat-masked reference), and for 74.0% of the HHV-6A complete genome (85.4% of the repeat-masked reference).

We produced *de novo* assemblies of our sequences and compared the variant calling reference-based and the one produced from aligning assemblies, obtaining an almost perfect overlap. We manually curated the few ambiguous variants and deposited the final sequences in Genbank.

### Inherited chromosomally integrated HHV-6 identification

The LCLs from the sequenced samples were tested for the presence of iciHHV-6. Following the method proposed by Sedlack *et al*.^25^, we performed droplet digital PCR (ddPCR) in order to quantify the absolute number of HHV-6 copies and human genome copy. If the virus was present in an inherited form, we would expect to have a copy of its genome per each cell. The results showed around 1 HHV-6 copy per cell (range: 0.92–1 copy/cell), supporting the inherited condition of these viruses. While very unlikely, the clonal expansion of a lymphocyte bearing integration of non-inherited form of HHV-6 cannot be excluded, as it would produce similar results in a ddPCR analysis.

### HHV-6A and HHV-6B species show different diversity patterns

We integrated all available HHV-6 complete sequences published up to the date of performing this study to our dataset and compared intra-species diversity values. A second data set, referred to as iciHHV-6, was built selecting only the proven congenital sequences. All diversity analyses were repeated in this subset obtaining similar results unless we indicate otherwise.

Overall variability was calculated and corrected for the different number of sequences in the data sets using the Watterson estimator^64^ Θ_w_ applied to the SNV density of each data set. The diversity within the A and B species resulted notably higher in the former. The ratio of the number of polymorphism was three-fold higher in HHV-6A compared to HHV-6B. The ratio was similarly higher in HHV6 in all genomic features analyzed (HHV-6A/HHV-6B ratios: exons = 3.12, introns = 2.73, UTR = 2.97, Unclassified = 2.89; iciHHV-6A/iciHHV-6B: exons = 3.60, introns = 4.51, UTR = 3.01, Unclassified = 2.93; all values expressed as ratio of Θ_w_ calculated on the number of SNV/bp). The absolute SNV density reflected this ratio, with 0.020 SNVs/bp for HHV-6A, compared to the 0.007 SNVs/bp of HHV-6B, and with 0.010 SNVs/bp for the iciHHV-6A, compared to the 0.003 SNVs/bp of the iciHHV-6B.

Within a single species, exon showed the highest level of constraints, where we measured the lowest SNV density, while introns showed the higher values (Supplementary Table S2).

Overall transition/transversion ratio (Ti/Tv) was high for both species (>2.3; Supplementary Table S3). The Ti/Tv ratio measured for both species were higher than for other *Herpesviridae* subfamilies, but similar to members of the *Bethaherpesvirus* subfamily such as Human herpesvirus 5 (HHV-5; a.k.a. Human cytomegalovirus (HCMV)), and Human herpesvirus 7 (HHV-7)^65^.

### Non-sense SNVs and the need of choosing a new reference sequence

SNVs that create or disrupt a stop codon can dramatically change the translation product and function of a gene. Only two SNVs of our datasets produced non-sense mutations. A stoploss in *U12* was present in all available HHV-6B sequences, except for the reference Z29, at the end of the gene’s coding region. In order to support this statement, the U12 stoploss mutation was validated by Sanger sequencing in all the newly produced HHV-6B sequences. In HHV-6A, a different stoploss in *U47* was detected in 4 of the 8 sequences (4 of the 5 iciHHV-6A sequences). These were the only mutations found affecting translation, hinting together with the lack of large structural variations to the presence of essentially intact genes and potentially functioning viruses, in agreement with Zhang *et al*.^66^.

It is remarkable that the non-sense variant in HHV-6B is only absent in the Z29 sequence. Additional evidences indicated that the Z29 is quite divergent from the rest of sequences; SNVs that are exclusive of the Z29 strain add up to 37% of the total number of SNVs in the HHV-6B comparable data set (337/905 SNVs), compared to the 15% (370/2442) of exclusive SNVs of the U1102 strain (HHV-6A reference) in the HHV-6A data set. The presence of stratification in HHV-6B populations could explain, to some extent, the divergence shown by Z29 but, importantly, the observation holds when considering only the strains with the same geographical origin.

### HHV-6 population structure: stratification, phylogeny and recombination

**Figure 2:**
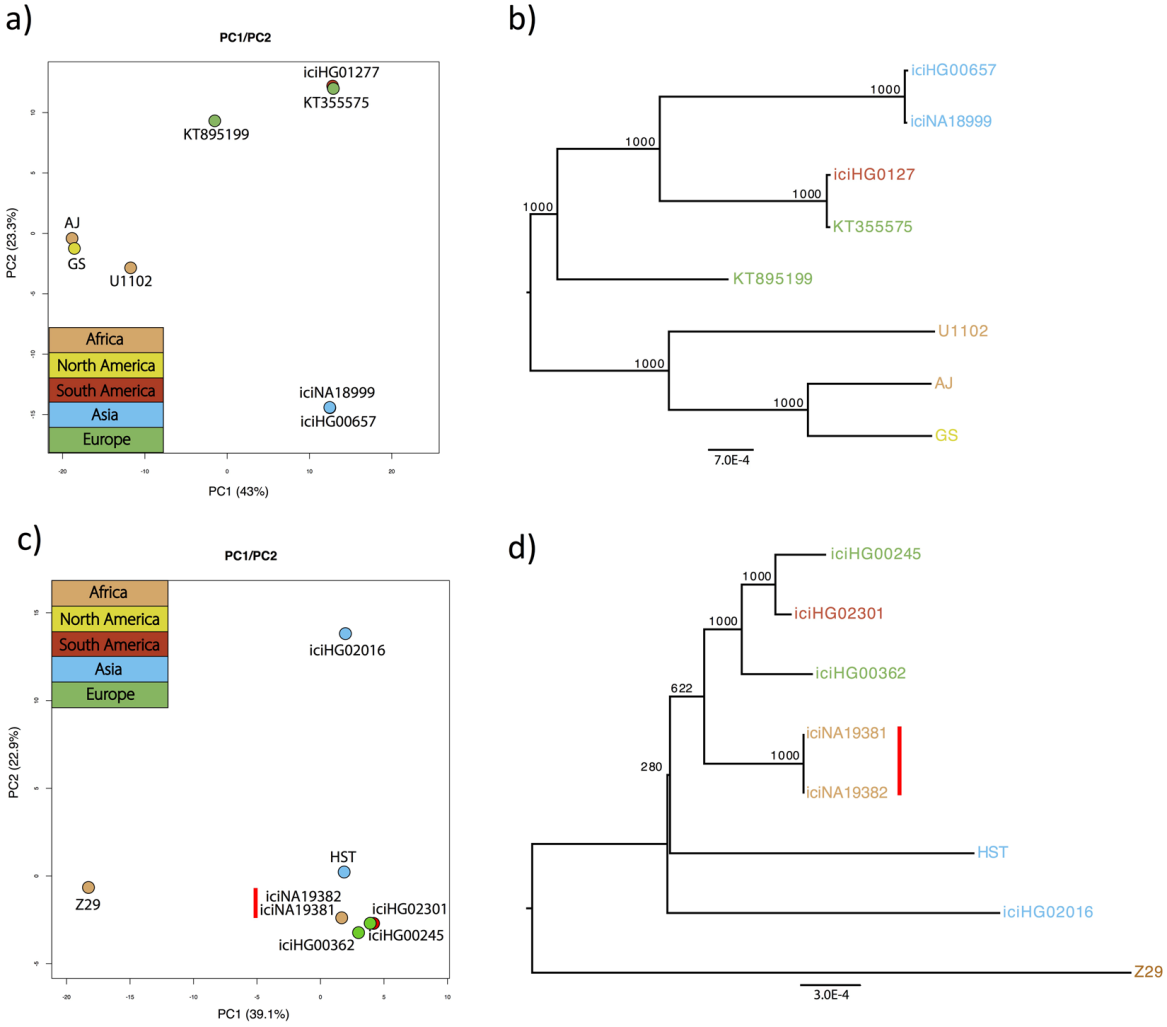
Geographical stratification in HHV-6. (**a)** PCA and (**b)** Neighbour joining tree, built using all the SNVs in HHV-6A strains. (**c)** PCA and (**d)** Neighbour joining tree, built using all the SNVs in HHV-6B strains. Individuals are color-coded based on the geographical origin. The vertical red line indicates the first-degree family members duo.

We performed Principal Component Analysis (PCA) separately in HHV-6A and -6B. In HHV-6A the Asian sequences cluster closely together when plotting the first two components (Fig. 2a), of which variation explained sums up to 66.3% of the total. While AJ and U1102, both of African origin (Gambia and Uganda respectively), cluster together, AJ falls also very close to the North American strain GS. This similarity is consistent with what had been reported by Tweedy *et al*. in the study where the AJ sequence was first published^67^. We observed a clear separation between Asian and African sequences explained by the first principal component, a pattern found also in the sister Herpesviridae Human herpesvirus 4 (HHV-4)^68,69^. While the two European sequences (KT355575 from UK and KT895199 from Germany or Czech Republic) appeared relatively close, the former clustered with the South American iciHG01277 sequence, a pattern that was evident also in HHV-6B, but that could be explained by the reduced cohort of this study. HHV-6B shows little sign of geographic stratification, and the first principal component, explaining 39.1% of the variance, mainly separates the Z29 reference sequence from the rest of the data set, while the second principal component, explaining 22.9% of the variance, separates the Vietnamese sequence iciHG02016. The other sequences cluster together, with iciNA19381 and iciNA19382 appearing completely overlapped (Fig. 2c), an expected result due to the first-grade relationship of the individuals from where these two sequences derived^70^.

We built NJ-phylogenetic trees for both HHV-6 species resulting in a highly robust tree for HHV-6A, where the two African strains clustered together, even though the North American GS strain remained the most similar sequence to AJ. The Asian cluster remains well supported and well separated from the other sequences (Fig. 2b). The separation between Asian and African strains is well defined in this analysis. The tree built on HHV-6B sequences, in contrast, shows little signs of population structure by geography. Only two of the same-origin sequences clustered together. These are the African iciNA19381 and iciNA19382, which comes from individuals with first-degree parent-son relationship^70^. Since the endogenous form is transmitted vertically, the two sequences are virtually identical, and are thus uninformative for this analysis. As seen in the PCA, the two European sequences do cluster with each other, but together to the South American one, a pattern that appeared less explicitly in HHV-6A. Finally, this tree confirmed the separation of the reference Z29 from the rest of HHV-6B sequences (Fig. 2d).

**Figure 3:**
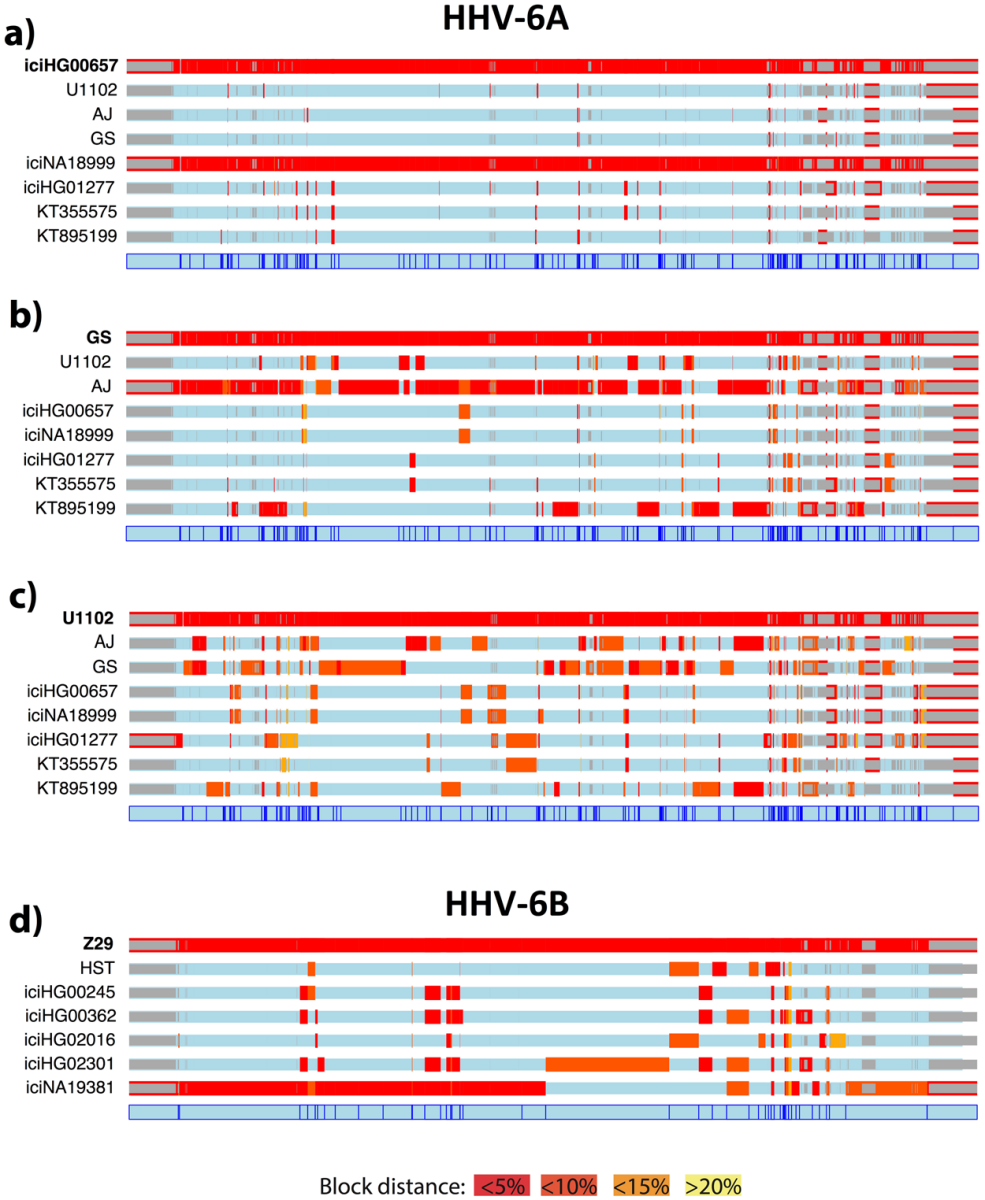
Recombination footprints. Recco analysis of the most cryptic HHV-6A (**a**–**c**), and HHV-6B (**d**) strains. The first row in each panel shows the analysed sequence, considered a recombination product of the rest of the species data set. The colour scale shows the proportional genetic distance, as shown in the appendix at the bottom of the figure. The masked regions of the genome are marked in grey.

The lack of a clear geographical pattern suggests the action of recombination. Analyses on recombination were conducted separately for the two species, for which every sequence has been analysed one at a time as the putative recombination product of the rest of sequences (see “Materials and methods” section). The analysis on the HHV-6A confirmed the genome-wide similarity of the Asian strains compared to the others, and the high whole genome identity between the GS and AJ strains. Interestingly, when the African U1102 sequence is analysed, patterns of similarities are evident with both the other African strain, AJ, and with the North American strain, GS. The latter presents similarity mainly with the two African sequences, sign of a possible African origin for this strain (Fig. 3a–c). The same analysis performed on the B species data set showed that Z29, the African sequence separated from all the other sequences in previous analysis, is very similar to the other African sequences compared to the rest of the data set (Fig. 3d). It should be noted that iciNA19381 and iciNA19382, the two congenital sequences derived from parent and son, have identical SNVs, so using them together in a block similarity analysis that scale itself on the minimum block distance would mask their putative similarity with the other sequences. To avoid this, we used only one of the two sequences, iciNA19381, as representative of the family. The region between 90–130 kbp is where most of the significant recombination breakpoints lie, which translates into stronger influence of this region in analysis such as PCA and phylogeny and, possibly, in concealing of clusters such as the African one. To confirm this, we constructed another tree excluding the 90–130 kb region. Remarkably, this resulted in the African sequences clustering together, as shown in Supplementary Figure S3.

The recombination analysis plots for each sequence of the data set are shown in Supplementary Figures S1 and S2.

### Selection footprints in coding genes: stronger selection constraints in HHV-6B compared to HHV-6A

**Figure 4:**
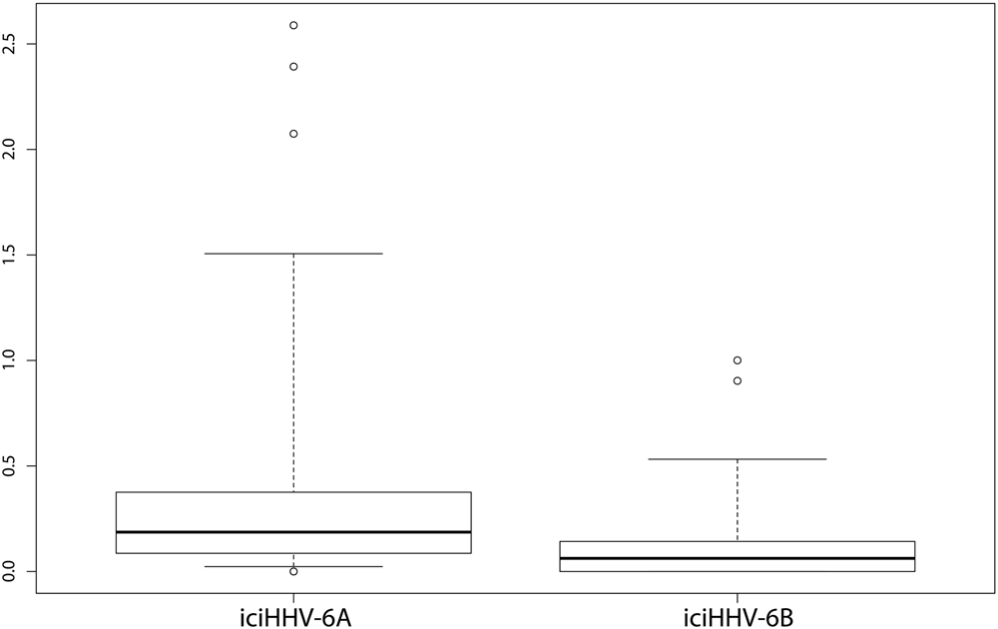
Selection footprints. The boxplots shows the gene ω values in iciHHV-6A and iciHHV-6B.

We measured rates of molecular evolution in protein coding genes in the iciHHV-6 data set for both species. Global dN/dS values (ω) were estimated for each gene, providing average ω values of 0.39 and 0.11 for iciHHV-6A and iciHHV-6B, respectively. While this value falls within the boundaries of the average human value for iciHHV-6B (average: 0.13, standard deviation: 0.11^71^), iciHHV-6A shows a remarkably higher value. The difference observed between iciHHV-6A and iciHHV-6B was significant (Fig. 4; Student’s T-test p-value, 0.00008).

Signals of positive or purifying selection were explored for single genes independently in both species. As described before, diversity within iciHHV-6B sequences was lower compared to iciHHV-6A. The number of SNVs in each gene was often low, leading to less robust estimations. We used different models in codeml to test for significantly high or low ω estimates, comparing the models using likelihood ratio tests (LRTs). We obtained the likelihood of a free-estimated ω with that obtained from an identical model but where ω was fixed at the average value reported above. This would identify an ω value that is at the tail of distribution. A second LRT was performed between the likelihood of the model leaving ω free for estimation, and the same model were ω was fixed to 1, a value considered a threshold to identify positive selection (Table 2). Two genes stood out for high dN/dS ratio in iciHHV-6B: *U90* and *U100*, with an ω value of 0.90, and 1.00 respectively. iciHHV-6A showed more diversity than its sister species, increasing the power to detect signatures of selection. There was no clear pattern of sequence constraints when observing the distribution of the ω values along the genome, with the exception of the IE-A region ω values tended to be higher. The highest ω values supported by LTR analysis was obtained for U95 (ω = 2.39), the only gene showing clear signs of positive selection. Signs of accelerated evolution were found in genes *U11*, *U24*, *U47* and *U90*, with ω estimates of 0.97, 1.00, 1.36, and 1.01 respectively (see Table 2). This is consistent with Dominguez *et al*. findings^4^, where *U90* and *U95* were also pointed out as the genes with highest Ka/Ks. Significantly low ω values were found in various genes: *U24A*, *U56*, *U63*, *U33*, *U51*, *U4*, *U39*, *U48*, here ordered *per* lower value of ω.

**Table 2:** Genes showing strong selection footprint. Genes with estimated ω significantly different from the average value of the species is shown. The LRT results between a model that estimates ω for each branch of the tree and the same model with ω fixed at the average value of the species, and fixed at 1, are reported.

Gene	Omega (ω)	LRT(est.ω VS avg ω)	LRT(est.ω VS ω = 1)
**iciHHV-6A**			
U11	0.9704	0.025957	0.986413
U24	1.3613	0.045580	0.986414
U47	1.0043	0.005118	0.986415
U90	1.0139	0.008566	0.986416
U95	2.3926	0.000001	0.003436
U4	0.0666	0.035232	3.40E-06
U24A	0.0001	7.06E-05	5.51E-08
U33	0.0232	0.007389	4.52E-06
U39	0.0799	0.027790	1.22E-06
U48	0.0860	0.003596	1.09E-09
U51	0.0597	0.020087	1.78E-06
U56	0.0001	0.045580	0.000595
U63	0.0001	0.004029	1.47E-05
**icIHHV-6B**			
U90	0.903	0.006543	0.998871
U100	1.000	0.027501	0.998871

## Discussion

In the present study we performed a genome-wide comparative analysis on HHV-6A and HHV-6B sequences. We report here nine new iciHHV-6 sequences derived from healthy 1KGP individuals. Using targeted genomic enrichment, we could generate deep-coverage iciHHV-6 genomes. These new sequences combined with previously published HHV-6 sequences, make it possible to perform phylogenetic, recombination and selection analysis. This data set has been used to assess variability within, and compare it between, HHV-6A and HHV-6B, and iciHHV-6A and iciHHV-6B. We have also explored geographical stratification and proposed a set of genes as candidates to be under trends of either accelerated evolution or positive, or purifying selection.

Mutation rates become an important factor when analysing virus variability, and its estimation would help to pinpoint the divergence time between species, and to explore the possibility of a single or multiples germ-line integration events. In our data set we presented the sequences of iciHHV-6B belonging to two individuals with a first-degree relationship (iciNA19381, iciNA19382), which could be used to infer the mutations fixed per generation. The two-sequence showed one single different SNV after variant calling, where iciNA19382 presents the mutation, while iciNA19381 does not. A Sanger sequencing of an amplicon covering the position of the SNV performed on both individuals showed that the mutation was indeed present in both. The family duo thus resulted identical when the SNVs were compared, precluding the estimation of a mutation rate. The lack of SNVs between the two sequences is nevertheless expected when considering the estimation of the mutation rate described for other herpesviruses^72,73^, and the relatively short length of the HHV-6 genome. More of such duos would make it possible to estimate a mutation rate per generation for iciHHV-6.

SNV densities were statistically significantly different between the A and B species, in both the whole HHV-6 data set and the iciHHV-6 subgroup, where the variation was homogeneously distributed along the genome. The difference in divergence is consistent with what has been reported by Tweedy *et al*. for iciHHV-6^74^.

Analysis made on SNV and their frequency distribution rose doubts on the convenience of keep using Z-29 sequence as a reference genome for HHV-6B; this sequence showed a disproportionate number of private mutations compared to the rest of sequences from the same or different continents. The HHV-6A reference strain instead (U1102), shows comparable number of private mutations. Non-sense mutations support this hypothesis, showing in our dataset that while in HHV-6A the stoploss in *U47A* is equally probable than the reference allele, the elongated form of *U12* in HHV-6B was missing only in Z29, indicating that the event could rather represent a stop-gain occurred in the reference strain than a stop-loss occurred in all other sequences. The change in *U12* length is notable, with the two coding regions of this gene fusing into one. *U12* encodes a G protein-coupled receptor involved ultimately in cellular response. No functional consequence of the change in length of this protein can be hypothesised due to the limited information available. An additional hint of the dissimilarity of the reference sequence Z29 can be found in the study by Stanton *et al*.^75^, in which two HHV-6B groups are described based on the variation within the *IE1* gene in 14 sequences. The two groups clustered around Z-29 or HST. Notably, the HST-like group was much larger than Z-29, including 10 of the 14 sequences of the data set. As a whole, and taking into consideration the whole set of published HHV-6B genomes, these evidences support that Z-29 is not a common sequence, and that HST, or any of our newly produced sequences, would be a better candidate to serve as reference sequence for HHV-6B.

Previous studies have interrogated putative HHV-6 genetic stratification^36,55,76^, but none of these studies ever attempted the analysis at a whole-genome scale. We found evidence of a solid Asian cluster in HHV-6A. The reference U1102 and the AJ strain, from Uganda and Gambia respectively, show strong relationship whereas the GS strain from North America is highly similar to AJ, as previously described^67^. Nevertheless, the African and Asian strains appeared well separated from each other, as described in the sister *Herpesviridae* Human herpesvirus 4^68,69^. The European sequences, from Germany or Czech Republic, and from UK, show low divergence, but the latter cluster deeply with the South American strain. This pattern could be driven by recombination, or be a consequence of the low resolution of our analysis with a small data set.

On the contrary, no clear geographical pattern was observed in HHV-6B, with the two European strains clustering together, but with the UK sample showing more similarity to the South American one. Nevertheless, the other European sample originates from Finland, a population that is known to segregate from the other population of the same continent^77,78^. The African family iciNA19381-iciNA19382 also clusters near the Europeans and South American sequences. Recombination events among strains from different geographic origin might be masking global signals of stratification. Recombination analysis indicated that the Z-29 sequence from Africa was the most similar to the other African sequence in a sizeable part of the evaluated genome, supporting the presence of an African component. Sample size is key in this kind of analyses and our low resolution precludes us from exploring in full detail the presence and structure of geographical stratification in these viruses.

Evidence of accelerated evolution was found in a higher number of genes in iciHHV-6A compared to iciHHV-6B. We listed the genes with strong footprints of a history of accelerated or negative selection. The two genes showing significant evidence of accelerated evolution in iciHHV-6B were *U90* and *U100*, for both of which the results of the LRTs showed significant differences between the estimated ω and the average for the species, but almost no sign of difference when the comparison was against the model using a fixed ω of 1 (Table 2). Both genes had already been described by Dominiguez *et al*.^4^ as presenting high dN/dS (>1) when comparing HHV-6A and HHV-6B reference sequences. The protein encoded for by *U90* is an Immediate-Early transactivator, putatively regulating RNA replication, transcription and modification during the first phase of HHV-6 active infection^4^. *U100* instead encodes for the gp82-gp105^79,80^, a glycoprotein related to CD46 substrate binding and fusion, and is thus involved in the different tropism of HHV-6 species. In iciHHV-6A, where selection footprints were more distinct due to the higher variability, evidence for signals of positive selection were obtained for *U95*, while *U24*, *U11*, *U47* and *U90* showed signs of accelerated evolution. Two of the five genes have already been detected as having high dN/dS values when comparing the HHV-6A and -6B references^4^ (U90, U95). In the same study, also *U54* and *U91* showed high dN/dS values, genes for which our estimated ω was high (>1.5), although in our set of sequences these high values were not statistically significant. *U24* is an important gene related to the host immune response, inhibiting T-cells activation^81^, and blocking early endosomal recycling^82^, thus minimizing immune recognition. U11 is instead a necessary antigenic protein for virion reconstitution^83^. Lastly, U95 is also a putatively important gene, having been shown to indirectly modulate cell death signals in HHV-6B^84^.

One of the most constrained genes in iciHHV-6A was expectedly *U48*, the gene encoding for the glycoprotein H of the gL-gH-gQ complex, and that is known to be conserved across the *Herpesviridae* family.

## Materials and Methods

### Individuals showing HHV-6 integration and variant identification

We downloaded Illumina-sequenced, paired-end reads from the individuals of the 1KGP Phase 3 non-mapping to the human genome reference (b37). We then mapped these reads against HHV-6A and B reference strains (NCBI accession numbers: NC_001664.2 and NC_000898.1 respectively) using Burrow-Wheelers Aligner^85^ (BWA) and setting the phred trimming quality threshold to 15. Only uniquely mapping reads were selected for HHV-6 genome reconstruction and comparative analysis. BAMs were masked for low-complexity and main repeats regions using NCBI annotations, RepeatMasker^86^, and Tandem Repeat Finder^87^. Duplicated reads were removed using the SAMtools package rmdup function^85^. SNVs and Insertion/Deletions callings were performed on the obtained alignments using VarScan 2^88^ set with default parameters. The resulting SNVs files have been used as known polymorphic sites for quality score recalibration of the alignments themselves through Genome Analysis ToolKit^89^ (GATK). Recalibrated files were re-mapped with the same settings, and depth of coverage was calculated using GATK’s DepthOfCoverage function. Individuals were considered as presenting HHV-6 latency when the median coverage along the whole masked virus genome was above or equal to 2.

HHV-6A and -6B typing was achieved by checking for species-specific variants in the U65 open reading frame. Starting from position 101,548 of the reference sequence U1102 coordinates, the HHV-6A species was identified by 5′-**T** TGT G**T**G TT**G** TTT T**A**-3′. The HHV-6B species was identified by 5′-**G** TGT G**C**G TT**A** TTT T**C**-3′ starting from position 102,848 of the reference sequence Z29 coordinates. In order to avoid incorrect species assignment, we mapped the reads belonging to each individual against both HHV-6A and HHV-6B species, and calculated the relative coverage ratios along the whole genome. We did not found any case with ambiguous variant assignment.

### Whole-genome target enrichment and repository upload

Available DNA samples belonging to 1KGP individuals presenting HHV-6 integration were retrieved from established blood-derived LCL at Coriell Institute for Medical Research (Camden, NJ, USA).

Illumina sequencing libraries were produced following Illumina TruSeq® DNA Library Prep LT protocol starting from 1 µg of the retrieved LCL DNA per sample. Library preparation was followed by target enrichment using custom RNA baits covering more than 99% of the two HHV-6 species genomes (SeqCap EZ Library SR, Nimblegen). The Genomic Unit of the Centre for Genomic Regulation (CRG, Barcelona, Spain) performed library preparation and target enrichment experiments. The resulting libraries were sequenced on an Illumina MiSeq System at Pompeu Fabra University (UPF, Barcelona, Spain) Genomic Core Facility using the 300-cycle MiSeq reagents kit V2 (Illumina), and base calling was performed using the MiSeq Reporter Illumina pipeline.

The sequences obtained by target enrichment were scanned for repeats longer than 500 bp, which were masked from the sequence and excluded from all analyses. The catalogue of repeats was obtained by merging to the NCBI reference sequences annotated ones the results of Repeat Masker, and Tandem Repeat Finder.

Reads were processed and mapped following the same protocols as above (see “Individuals showing HHV-6 integration and species identification” section). Using the information provided by the paired-end mapping we interrogated large structural variation (such as insertions or deletions, inversions and duplications). Inversions: Identifying clustered mate reads with same orientation, with depth of coverage falling within 3 standard deviations from the mean of the sample. Insertions/Deletions: Insert size distributions were calculated for each individual. Insert size peaks separated from the average insert size by at least 3 standard deviation were considered as putative large deletions or insertions. Putative insertions were scanned for the presence of high-density SNV clusters. Large insert sizes due to circularity of the viral genomes were manually excluded. Duplications: Identifying regions with depth of coverage doubling the median value in windows of 1000 bp (shift of 100 bp).

Genomic GC content was calculated in the same 1000 bp sliding windows mentioned above, using the GC function of the R package Seqinr^90^.

### Inherited chromosomally integrated HHV-6 identification

The samples were tested for inherited HHV-6 integration presence using digital droplet PCR (ddPCR). The experiments followed the method proposed by Sedlack *et al*.^25^, starting from 150 ng of sample DNA per reaction.

### *De novo* assemblies

Contigs for each individual were produced starting from the raw sequencing reads using Discovar *de novo* assembler^91^. The contigs were filtered for a minimum length of 1000 bp, and their reference-based coordinates were determined aligning them to the species reference genome using Geneious V5.6.7^92^. Variants not supported by the reference-based variant calling were manually corrected. A final reference-based scaffolding was achieved using Ns for gaps, and annotations were transferred from the specie reference sequence to the assemblies using RATT^93^.

### Variability analysis

Variant calling was performed using VarScan 2, setting a minimum base coverage of 20, with the criterion that the loss of the callable genome was less than 15%. Variants were additionally filtered for extreme allele balance (frequency < 5%). Transition/transversion ratios were calculated using vcftools^94^.

Diversity values between species were obtained by counting the number of SNV divided by masked feature length, and correcting for the number of sequences with the Watterson estimator.

Variability across the genome was calculated by counting SNVs in 10 Kbp bins of the repeat-masked genomes.

Genomic intervals of the two viruses that were common across samples in terms of ‘callability’ were calculated and all comparative analysis (*i*.*e*. PCA, phylogenetic trees generation, recombination, variability patterns and selection footprints) were performed using this common set of positions.

### Sanger sequencing SNV validations

The first-degree relationship individuals present in our data set, iciNA19381 and iciNA19382, showed a single SNV of difference after the variant calling. Being the locus one with decreased base quality compared to the neighbouring regions, we checked the status of the SNV sequencing through Sanger technology the position in both individuals.

A 500 bp amplicon was amplified in 25 µl reactions using the primers FamMutFWD = 5′- GGACATCTCTTTGTTGTGTGCC-3′ and FamMutREV = 5′-GGCTGGTATTAGAACAATTAGGACA-3′ (20 mM Tris HCl, 50 mM KCl, 2 mM MgCl2, 200 µM dNTPs, 1.5 U BioTherm Taq DNA Polymerase (GeneCraft), 900 nM each of primer, 50ng sample). The PCR reaction was purified using QIAquick Purification Kit, and run on a 2% agarose gel to control the amplicon length. Each amplicon was then sequenced separately two times, one for each primer. This was performed following the Big Dye Terminator v3.1 kit (Thermo Fisher) guidelines, starting from 10 ng of the purified PCR reaction.

Following the same protocol, the stoploss found in all newly generated sequences in the U12 ORF was validated. The primers were: U12stlssFWD = 5′-GTAAGCAGACCGAAAGTAAAAC-3′ and U12stlssREV = 5′-TAGAGAAACAATGTACCTGTGG-3′.

### Population structure: stratification, phylogeny and recombination

All HHV-6 complete sequence published at the beginning or during the performance of this project were added to our newly assembled sequences for a comprehensive comparative analysis, for a total of 8 sequences for each HHV-6 species. The reduced list of HHV-6 sequences included U1102^95–97^ (GenBank Accession number: NC_001664.2), GS^98^ (GenBank Accession number: KC465951.1), AJ^67^ (GenBank Accession number: KP257584.1), KT895199^74^ (GenBank Accession number: KT895199.1) and KT355575^74^ (GenBank Accession number: KT355575.1) for the A species, and Z29^4^ (GenBank Accession number: NC_000898.1) and HST^5^ (GenBank Accession number: AB021506.1) for the B species.

PCA was performed using the Prcomp function in Stats R package, and plotted using basic R plot functions^99^. Partial genome sequences were aligned with MAFFT^100^ and phylogenetic analysis were performed on transformed sequences obtained by substituting each identified SNV to the correspondent base in the reference genomes. Because members of the Herpesviridae family are well known to undergo homologous recombination among viromes^101–103^, the phylogenetic trees were built using neighbour joining algorithms, with no assumptions regarding recombination. Using the MEGA7 software^104^, the alignments were read, the genetic distances and, the neighbour-joining trees were computed, and the bootstrap values calculated. Trees were rooted at midpoint, and bootstrap analysis were set at 1000 replicates per tree. The trees were visualized using FigTree. Recombination breakpoint calculations were performed with Recco^105^; genetic identities between predicted recombinant blocks and plots were performed as in Santpere *et al*.^68^.

### Selection on coding genes

The phylogenetic trees generated for the enriched genomes in the previous phase were used to calculate dN/dS values for each gene of the two viral strains using the codeml program implemented in PAML package^106^, together with the sequences available in NCBI for the published strains. codeml was set to run applying M0 model (Nsites = 0) with ω estimated for every branch of the tree, and the same model fixing ω at 1 and at the average of the estimated ω values for every gene. Genes that presented no SNV were excluded from the analysis of the diversity between species in ω distributions, leading to the exclusion of 9 genes in iciHHV-6A, and 18 in iciHHV-6B.

We obtained maximum likelihoods values for every gene using the aforementioned different models and LRTs was used to compare them. P-values were calculated from the cumulative chi-square distribution with a number of degree of freedom equal to the difference in free parameters in the model (1 in our case). In order to correct for multiple comparisons and limit the false discovery rate, we applied Benjamini & Hochberg method^107^ to our p-values using the P.adjust function of the Stats package bundled with R. The comparison between ω distributions in iciHHV-6A and iciHHV-6B was plotted using the Adjbox function of the Robustbase R package^108^.

SNV functional annotation, including coding stop-gain/loss, was performed using Annovar^109^.

### Data availability

Target enrichment Illumina raw reads are available at the Short Read Archive under the BioProject ID: PRJNA412600. Assembled sequences are available at GenBank and identified by the accession number MG894368-76.

### Ethical approval and informed consent

The present study involves human genotyping data made publically available by the 1KG project with no need of ethics approval. It also involved LCL from Coriell Institute, to obtain the samples from Coriell we produced the required Statement of Research and Assurance Form for Biomaterials approved by the Institutional Official of the Pompeu Fabra University.

## Electronic supplementary material

Suplementary information
