# Author Correction: Whole genome diversity of inherited chromosomally integrated HHV-6 derived from healthy individuals of diverse geographic origin

**DOI:** 10.1038/s41598-018-24603-9

**Published:** 2018-04-17

**Authors:** Marco Telford, Arcadi Navarro, Gabriel Santpere

**Affiliations:** 1 Institute of Evolutionary Biology (UPF-CSIC), Departament de Ciències Experimentals i la Salut, Universitat Pompeu Fabra, PRBB, Barcelona, Catalonia Spain; 2 National Institute for Bioinformatics (INB), PRBB, Barcelona, Catalonia Spain; 3 Institució Catalana de Recerca i Estudis Avançats (ICREA), PRBB, Barcelona, Catalonia Spain; 4 Center for Genomic Regulation (CRG), PRBB, Barcelona, Catalonia Spain; 5 Department of Neuroscience, Yale School of Medicine, New Haven, CT 06510 USA

Correction to: *Scientific Reports* 10.1038/s41598-018-21645-x, published online 22 February 2018

The Acknowledgements section in this Article is incomplete.

“This work was supported by Ministerio de Ciencia e Innovación, Spain (BFU2012-38236 and BFU2015-68649-P to A.N.), by Direcció General de Recerca, Generalitat de Catalunya (2014SGR1311 and 2014SGR866), by the Spanish National Institute of Bioinformatics (PT13/0001/0026) and the REEM (RD12/0032/0011, RD16/0015/0017) of the Instituto de Salud Carlos III and by FEDER (Fondo Europeo de Desarrollo Regional)/FSE (Fondo Social Europeo).”

should read:

“This work was supported by Ministerio de Ciencia e Innovación, Spain (BFU2012-38236 and BFU2015-68649-P (MINECO/FEDER, UE) to A.N.), by Direcció General de Recerca, Generalitat de Catalunya (2014SGR1311 and 2014SGR866), by the Spanish National Institute of Bioinformatics (PT13/0001/0026) and the REEM (RD12/0032/0011, RD16/0015/0017) of the Instituto de Salud Carlos III and by FEDER (Fondo Europeo de Desarrollo Regional)/FSE (Fondo Social Europeo).”

