# Supplementary material for: Whole genome diversity of inherited chromosomally integrated HHV-6 derived from healthy individuals of diverse geographic origin
Source: Sci Rep. 2018 Feb 22;8:3472. doi: 10.1038/s41598-018-21645-x (PMC5823862; doi:10.1038/s41598-018-21645-x)
Supplement: Supplementary file 1 — Suplementary information [file 41598_2018_21645_MOESM1_ESM.pdf]

SUPPLEMENTARY MATERIAL

Whole genome diversity of inherited chromosomally integrated HHV-6 derived from healthy individuals of diverse geographic origin

Marco Telford[1], Arcadi Navarro[1,2,3,4,*], Gabriel Santpere[1,5,*]
* arcadi.navarro@upf.edu (AN)
* gabrielsantperebaro@gmail.com (GS)

[1] Institute of Evolutionary Biology (UPF-CSIC), Departament de Ciències Experimentals i la Salut, Universitat Pompeu Fabra, PRBB, Barcelona, Catalonia, Spain
[2] National Institute for Bioinformatics (INB), PRBB, Barcelona, Catalonia, Spain
[3] Institució Catalana de Recerca i Estudis Avançats (ICREA), PRBB, Barcelona, Catalonia, Spain
[4] Center for Genomic Regulation (CRG), PRBB, Barcelona, Catalonia, Spain
[5] Department of Neuroscience, Yale School of Medicine, New Haven, CT 06510, USA

SUPPLEMENTARY MATERIAL

Supplementary Figure S1 - **Recco analysis of all HHV-6A strains**. The first row in each panel shows the analysed sequence, considered a recombination product of the rest of the type data set. The colour scale shows the proportional genetic distance, as shown in the appendix at the bottom of the figure. The masked regions of the genome are marked in grey. The figure follows in the next page.

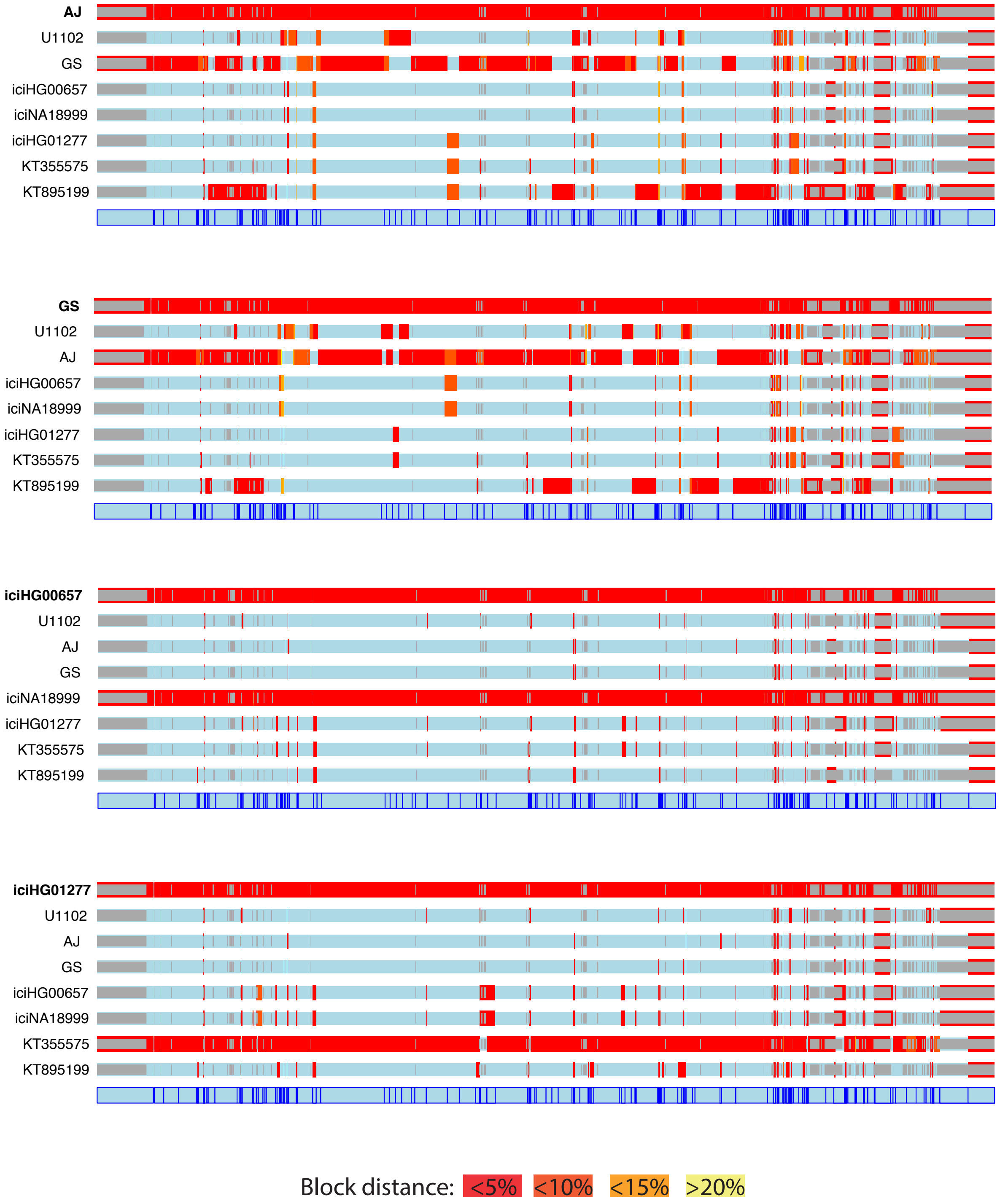

Supplementary Figure S1 – **Recco analysis of all HHV-6A strains**. The first row in each panel shows the analysed sequence, considered a recombination product of the rest of the type data set. The colour scale shows the proportional genetic distance, as shown in the appendix at the bottom of the figure. The masked regions of the genome are marked in grey.

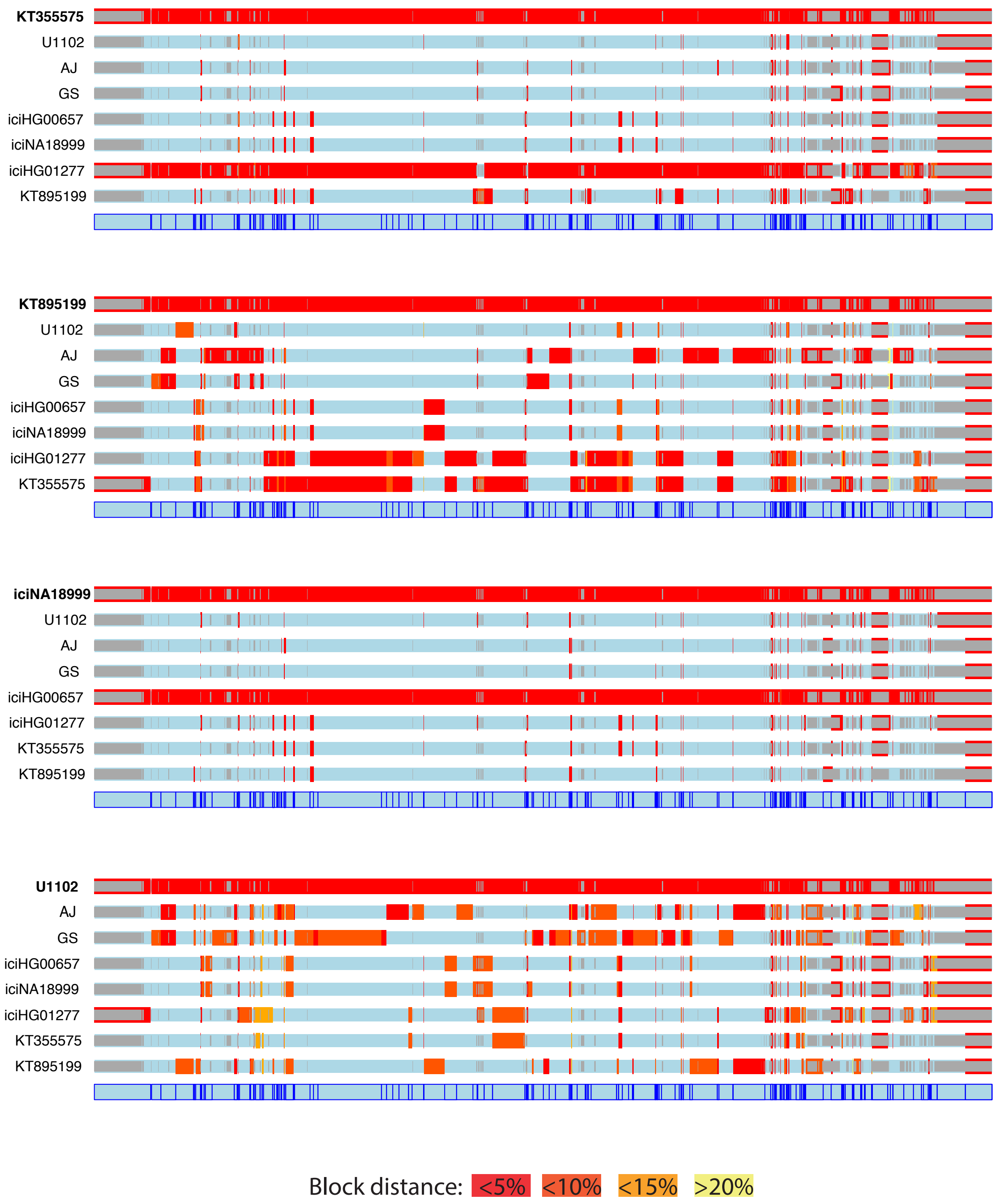

Supplementary Figure S2 – **Recco analysis of all HHV-6B strains**. The first row in each panel shows the analysed sequence, considered a recombination product of the rest of the type data set. The colour scale shows the proportional genetic distance, as shown in the appendix at the bottom of the figure. The masked regions of the genome are marked in grey. The figure follows in the next page.

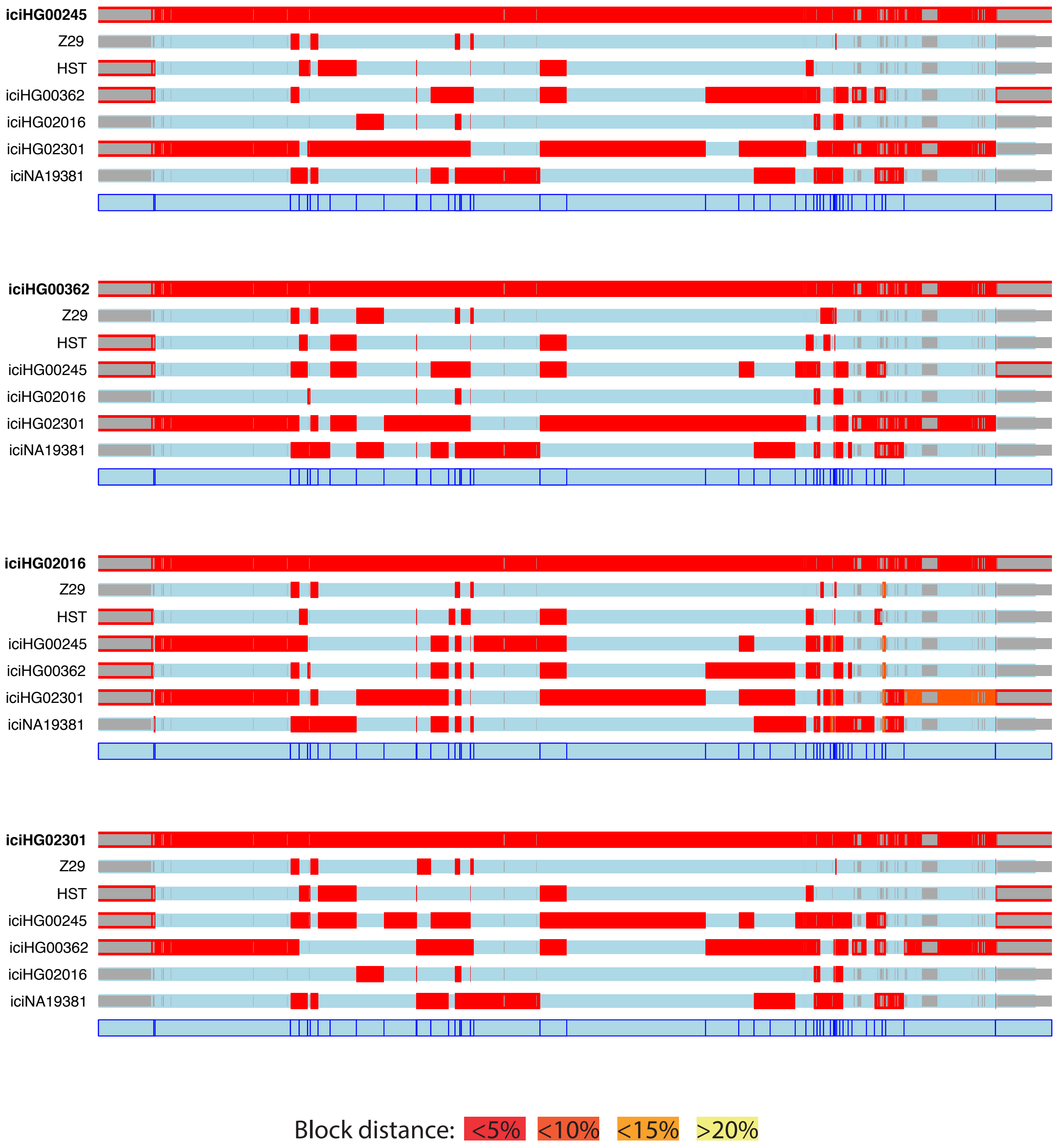

Supplementary Figure S2 – **Recco analysis of all HHV-6B strains**. The first row in each panel shows the analysed sequence, considered a recombination product of the rest of the type data set. The colour scale shows the proportional genetic distance, as shown in the appendix at the bottom of the figure. The masked regions of the genome are marked in grey.

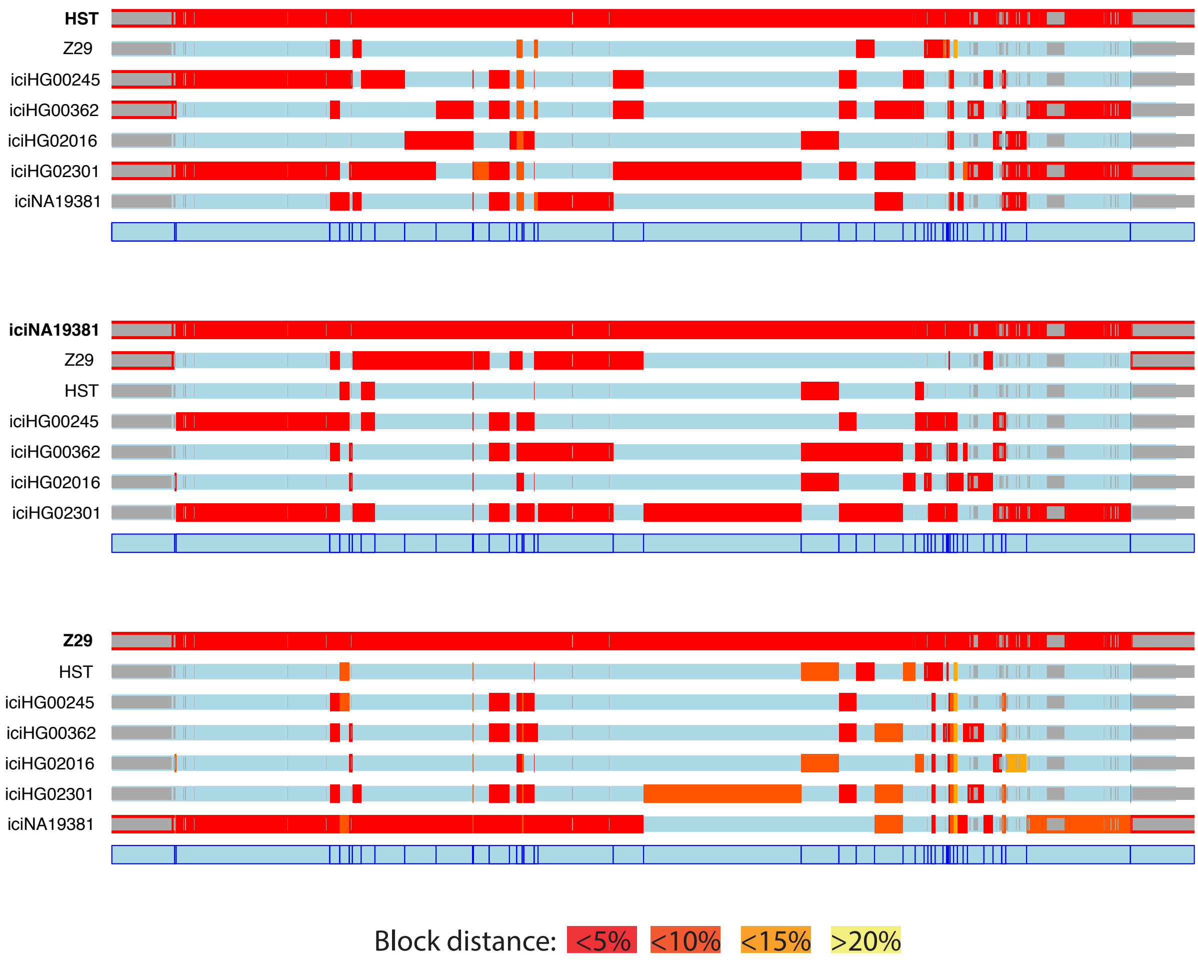

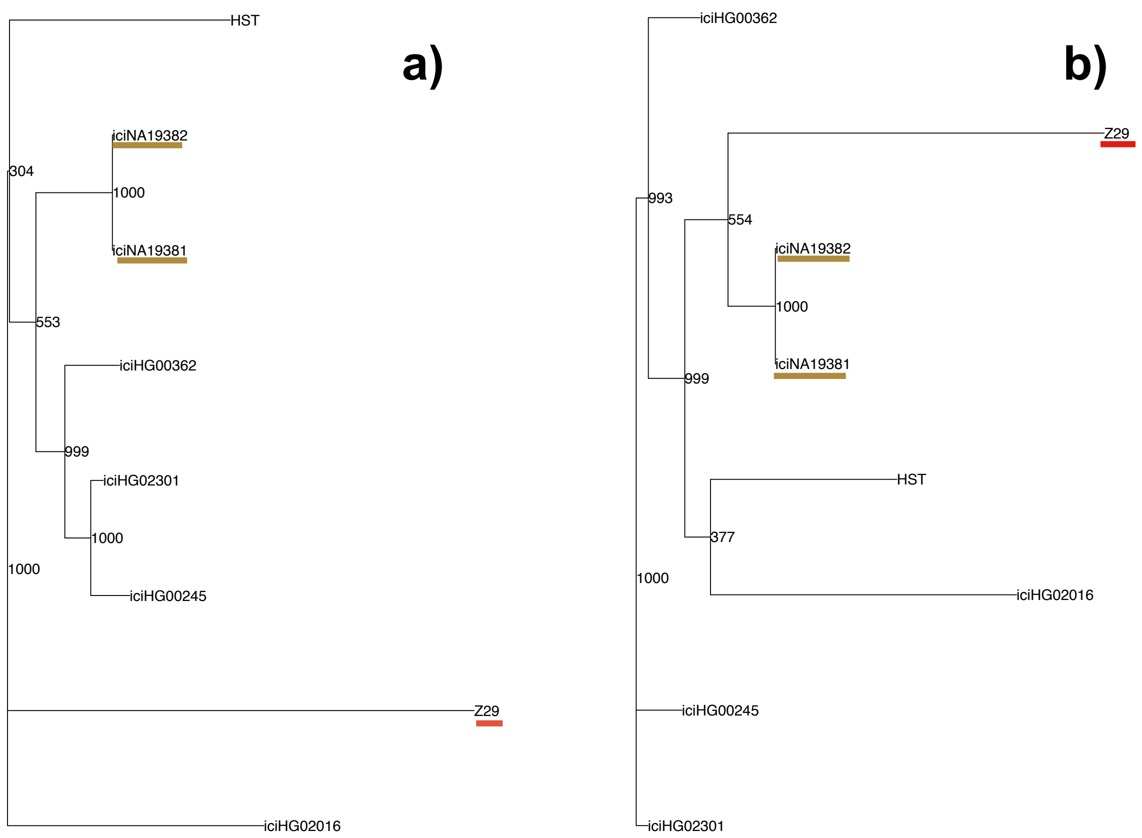

Supplementary Figure S3 – **African component in HHV-6B**. Unrooted neighbour-joining trees of the HHV-6B sequences built on a) whole genomes b) whole genomes excluding the region between coordinates 90000-130000. The African reference is underlined in red, while the other African sequences are underlined in orange. As expected from recombination analysis, when the region with the higher recombination breakpoints is taken away from the analysis, the African cluster becomes visible in the phylogenetic tree.

a)

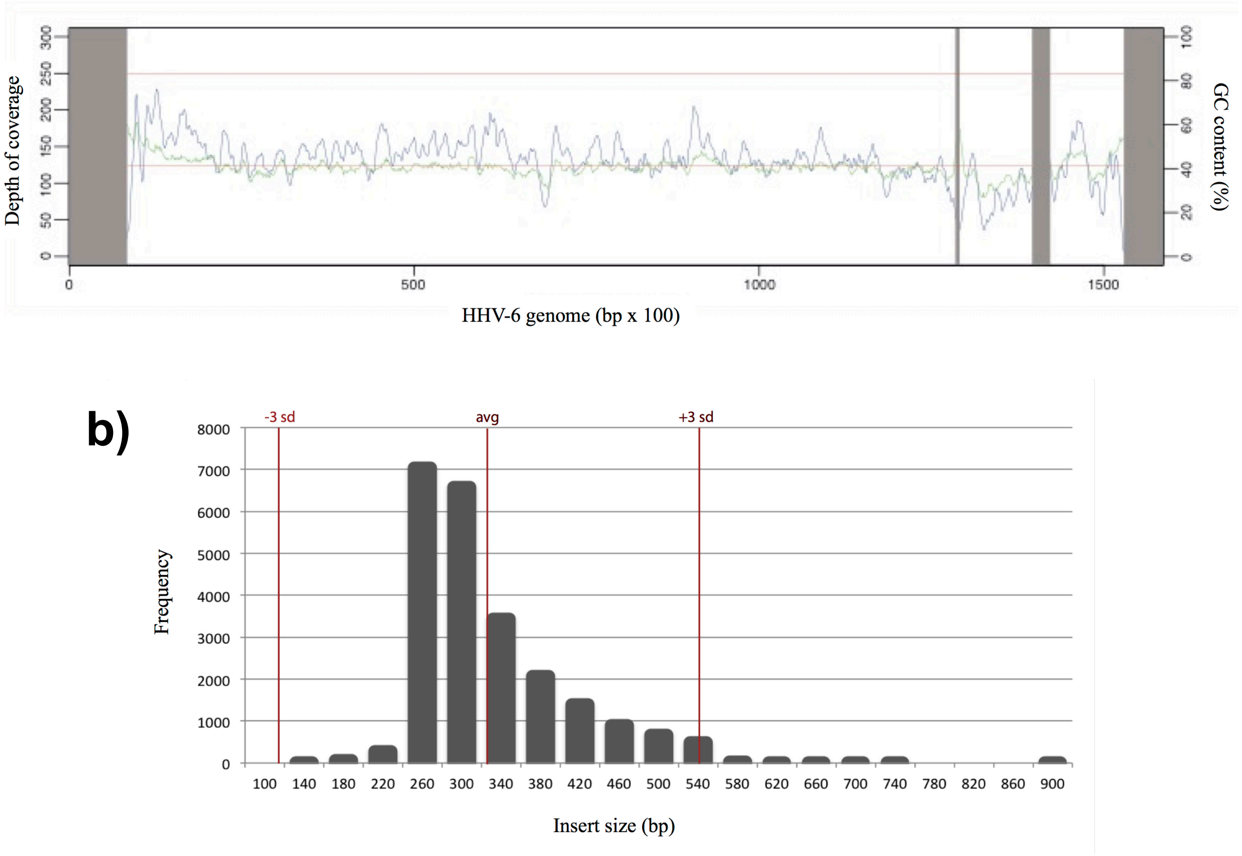

b)

Supplementary Figure S4 – **Large structural variation scans for an exemplifying individual (iciHG00245). a) Individual coverage along the genome**. Depth of coverage along HHV-6 genome calculated for 1000 bp sliding windows (100 bp steps). The average depth of coverage value, and its double, are shown by the lower and upper red lines respectively. Masked repeats are shown as grey boxes. A putative large duplicated region would show a depth of coverage value in the vicinity of the upper red line. **b) Insert size distribution and frequency**. The reds vertical lines indicate the average value (central line), and +/- three standard deviation from it.

HHV-6A		
**Start**	**End**	**ID**
0	8087	DR
151234	159322	DR
127496	128181	R1
131021	132254	R2
137880	140965	R3

HHV-6B		
**Start**	**End**	**ID**
0	8793	DR1
153321	162114	DR2
9314	9510	R0
129045	129681	R1
133500	133863	R2A
133981	134076	R2B
140081	142691	R3

Supplementary Table S1 - **Large annotated repeats per variant and their coordinates.**

Strain	Exons	Introns	UTRs	Others
**HHV-6A**	6,96	14,74	10,10	8,40
**HHV-6B**	2,23	5,41	3,40	2,91
**CiHHV-6A**	4,24	11,78	6,27	5,72
**CiHHV-6B**	1,18	2,61	2,08	1,95

Supplementary Table S2 – **SNV densities corrected through the Watterson estimator.**

Subfamily	Species	No. of Strains	Ti/Tv
Alphaherpesvirus	HHV-1 (HSV-1)	26	1.63
	HHV-2 (HSV-2)	2	1.29
	HHV-3 (VZV)	46	2.01
Betaherpesvirus	HHV-5 (HCMV)	124	2.53
	HHV-6A	8	2.79
	HHV-6B	8	2.40
	CiHHV-6A	5	2.53
	CiHHV-6B	6	2.72
	HHV-7	2	2.80
Gammaherpesvirus	HHV-4 (EBV)	9	1.36
	HHV-8 (KSHV)	3	1.05

Supplementary Table S3 - **Average transition/transversions ratio in human herpesvirus.** Transition/transversion ratios of herpesvirus species other than HHV-6 derived from Sijmons *et al*. 2015[1].

BIBLIOGRAPHY

1. Sijmons, S. *et al.* High-Throughput Analysis of Human Cytomegalovirus Genome Diversity Highlights the Widespread Occurrence of Gene-Disrupting Mutations and Pervasive Recombination. *J. Virol.* **89,** 7673–7695 (2015).
